# Swallowing Artificial Teeth.—Death and Autopsy

**Published:** 1873-11

**Authors:** E. D. Smith


### ARTICLE V.

### *Swallowing Artificial Teeth.—Death and Autopsy.*

BY E. D. SMITH, M. D.

Some years since, in the month of May, I received a telegram to go immediately to the western part of the State of New Jersey, to see a patient whom report said was dying. Four hours' ride by rail brought me to the bedside of the sick one, who proved to be a young married lady, Elizabeth S———, twenty-two years of age, whom I had known from youth.

She was greatly emaciated; a cold, clammy sweat stood upon the surface of her skin, which was white as snow; her bright black eyes were deeply sunken, with other marked symptoms of approaching dissolution. With a tearful eye and tremulous voice she gave me a history of her case, as follows: Four years previous she was married, with bright prospects for a happy life, but two weeks subsequently the young bride was a widow. Her husband missed his step and fell from a car in motion, and the entire train passed over his body, killing him instantly. The shock upon the nervous system was terrific, and from it she never fully rallied. Two years subsequent to this, she was again married, and one year of happy married life had passed when she gave birth to a fine male child during her labor, which was severe and protracted. Puerperal convulsions supervened. This complication her attending physician thought advisable to treat with copious blood-letting. The last convulsion she had, from the description given me by her mother, was terrific, lasting full twenty minutes. During this severe paroxysm she struggled fearfully, and from all appearances was choking to death. The physician who was present ordered the nurse to give her a spoonful of some fluid medicine he had just prepared. The effort to swallow this caused her to become fairly black in the face, strangling as though something was pressing upon the epiglottis, so as to entirely prevent the air from entering the lungs. During this exciting and dangerous condition, which lasted for near a minute, the patient placed her hands to her throat, as if endeavoring to tear it open; when, after an almost superhuman effort, relaxation ensued and respiration was resumed. The convulsions gradually decreased and finally ceased entirely, when her reason was again restored. Her throat now became very sore and inflamed, as though something rough had passed through and irritated it, and felt as though a foreign substance was lodged there.

When consciousness was fully restored she said to her mother: "Where are my teeth? I fear I have swallowed them!" referring to a partial set of four teeth, which had been inserted by a gold clasp to the molar teeth a short time previously. Her mother smiled and said: "No, my child, this can not be; it would be quite impossible for you to swallow them;" but the teeth could nowhere be found.

Her physicians, two of whom were in attendance, came in soon after. She told them her suspicions, for which she received only their ridicule. They informed her that it was an impossibility. Thorough search was then made for the teeth, but they could nowhere be found. The doctors laughed at her "whim," as they called it, and *absolutely refused to make an examination of her throat* (though urged to do so by the patient,) to see if it was lacerated or inflamed, and to ascertain by ocular evidence if her false teeth were there.

The patient remained it this condition for *three* days, her food in the mean time being fluids only. At the end of this time, by order of her physician, she ate a small piece of beefsteak. The act of swallowing this solid food pressed upon the teeth, which were at this time lodged in the pharynx, and by the peristaltic motion of the œsophagus, the portion of food, teeth and all, were carried down to a point near the cardiac orifice. This point, it seems, they could not pass, the hook of one end of the clasp to which the teeth were attached penetrating the œsophagus and remaining in this position.

For three months this patient lived in this condition, but gradually becoming weaker and weaker, and nearing the confines of her narrow home. Her physician was in daily attendance, and in the meantime was treating her for indigestion, general debility, incipient phthisis, etc., etc.; he always strongly and persistently denying the presence of the teeth, and the impossibility of her swallowing them.

It was at this stage of her misfortune (I will not say disease, for such she had not) that I was called in council. After hearing the history of her case, and learning all the facts connected with it, I made a thorough examination, and before concluding my investigations, satisfied myself that her suspicions were well founded; that her false teeth had in reality been swallowed; that it was this body that had nearly strangled her at the moment; that by giving her the liquid and the food already mentioned, it had been at once moved down so low as no longer to keep up the spasm of the muscles of the throat, which, otherwise, would have caused suffocation ; that the teeth were now low down in the œsophagus, and probably near the cardiac orifice, and were the real and only cause of her sickness.

I candidly expressed my convictions to the attending physician ; and for thus asserting what I honestly believed, I received from the doctor only insult, centempt, and derision ; and was sneeringly asked by him how it was possible for any one to swallow even a partial set of false teeth? and was told that I was old enough, and had had experience enough, to know better. He said it was simply ridiculous for me even to suggest a thing so utterly impossible.

I replied, that the probabilities were that the patient would live but a short time, and a *post-mortem* examination would settle the question between us. To this proposition he readily assented ; and I left the patient in his hands and returned to the city. Two weeks subsequently the physician and myself were again face to face beside the lifeless body, I insisting upon the correctness of my first impressions, and he as strongly maintaining his previously expressed opinion, that her *teeth* were not there. The autopsy was made; and this, ladies and gentlemen, was the result of the examination of this exceedingly interesting case. [The doctor here exhibited the plate and teeth to the members of the academy.] You will perceive the teeth are four in number ; and that they were lodged in the œsophagus, about two thirds the distance from the pharynx to the cardiac orifice. The clasp you see on one end of the plate had pressed against the œsophagus with such force that it caused first inflammation and ultimately ulceration ; the clasp penetrating the side of the œsophagus forming a hook, which prevented the teeth from entering the stomach, where possibly they might have passed the alimentary canal, and thus have saved her life. Now I hesitate not to say, that this patient was sacrificed by the obstinacy of the doctor in refusing to examine the case, when his art or skill might have been of service. This omission of duty on his part cost this beautiful young wife and mother her life. Had he thoroughly examined the patient's throat, after the convulsions had ceased, he would have discovered the teeth in the pharynx, where but little skill would have been required to remove them—*N. Y. Med. Review.*

---

